# Investigation of J-Aggregates of 2,3,7,8,12,13,17,18-Octabromo-5,10,15,20-tetrakis(4-sulfonatophenyl) Porphyrin in Aqueous Solutions

**DOI:** 10.3390/nano13212832

**Published:** 2023-10-26

**Authors:** Balkis Abdelaziz, Mariachiara Sarà, Sahbi Ayachi, Roberto Zagami, Salvatore Patanè, Andrea Romeo, Maria Angela Castriciano, Luigi Monsù Scolaro

**Affiliations:** 1 Department of Mathematical and Computer Sciences, Physical Sciences and Earth Sciences, University of Messina V.le F. Stagno D’Alcontres, 31, 98166 Messina, Italy; 2 Laboratory of Physico-Chemistry of Materials (LR01ES19), Faculty of Sciences, Avenue of the Environment University of Monastir, Monastir 5019, Tunisia; 3 Department of Chemical, Biological, Pharmaceutical and Environmental Sciences, University of Messina V.le F. Stagno D’Alcontres, 31, 98166 Messina, Italy

**Keywords:** porphyrins, aggregation, spectroscopic investigation, kinetic, DFT analysis

## Abstract

The highly distorted water-soluble 2,3,7,8,12,13,17,18-octabromo-5,10,15,20-tetrakis(4-sulfonatophenyl)porphyrin (Br_8_TPPS_4_^4−^) is readily protonated under acidic pH, forming the diacid H_2_Br_8_TPPS_4_^2−^ and subsequently the zwitterionic H_4_Br_8_TPPS_4_, which eventually evolves into J-aggregates. These latter species exhibit a relevant bathochromic shift with respect to the monomer with a quite sharp band due to motional narrowing. The depolarization ratio measured in resonant light scattering spectra allows estimating a tilt angle of ~20° of the porphyrins in the J-aggregate. The kinetic parameters are obtained by applying a model based on the initial slow nucleation step, leading to a nucleus containing m monomers, followed by fast autocatalytic growth. The *k_c_* values for this latter step increase on decreasing the acid concentration and on increasing the porphyrin concentration, with a strong power-law dependence. No spontaneous symmetry breaking or transfer of chirality from chiral inducers is observed. Both Atomic Force Microscopy (AFM) and Dynamic Light Scattering (DLS) point to the presence, in both the solid and solution phases, of globular-shaped aggregates with sizes close to 130 nm. Density functional theory (DFT) calculations performed on simplified models show that (i) upon protonation, the saddled conformation of the porphyrin ring is slightly altered, and a further rotation of the aryl rings occurs, and (ii) the diacid species is more stable than the parent unprotonated porphyrin. Time-dependent DFT analysis allows comparing the UV/Vis spectra for the two species, showing a consistent red shift upon protonation, even if larger than the experimental one. The simulated Raman spectrum agrees with the experimental spectrum acquired on solid samples.

## 1. Introduction

Aggregation of porphyrins is a well-documented phenomenon [1,2]. The large aromatic extension of the porphyrin ring is responsible for π-stacking among adjacent units when the molecules are forced from good to bad solvent conditions [3,4,5]. In this respect, many amphiphilic porphyrins have been synthetized by properly introducing hydrophilic pendant groups into their periphery [6,7,8]. Porphyrins with charged groups on the meso substituents are usually soluble in aqueous solutions, but depending on the degree of substitution, they can display interesting aggregation phenomena [9,10,11]. In this context, porphyrins with sulphonic groups on the meso-phenyl rings are studied in depth, since they could lead to the formation of J- and H-aggregates [12,13,14]. These species exhibit edge-to-edge or face-to-face arrangement of their electronic transition moments, respectively, leading to bathochromically or hypsochromically shifted absorption bands, in line with the exciton splitting theory of Kasha [15]. In the case of sulphonated porphyrins, J-aggregates display a quite large displacement of the B-band (up to 55 nm), with a concomitant sharpening due to motional narrowing [16]. In particular, the symmetrically substituted tetrakis-(4-sulfonatophenyl)porphyrin (TPPS_4_) has received much attention as, depending on the experimental conditions (concentration, pH, ionic strength and presence of cationic species), it aggregates in a variety of different mesoscopic structures [17,18,19,20,21,22,23]. Chirality can be transferred into these aggregates through chemical [24,25,26,27,28] or physical bias [29,30,31,32,33,34]. Also, spontaneous symmetry breaking occurs in the absence of any apparent chiral bias, under kinetic control and using specific mixing protocols [35].

**Scheme 1:**
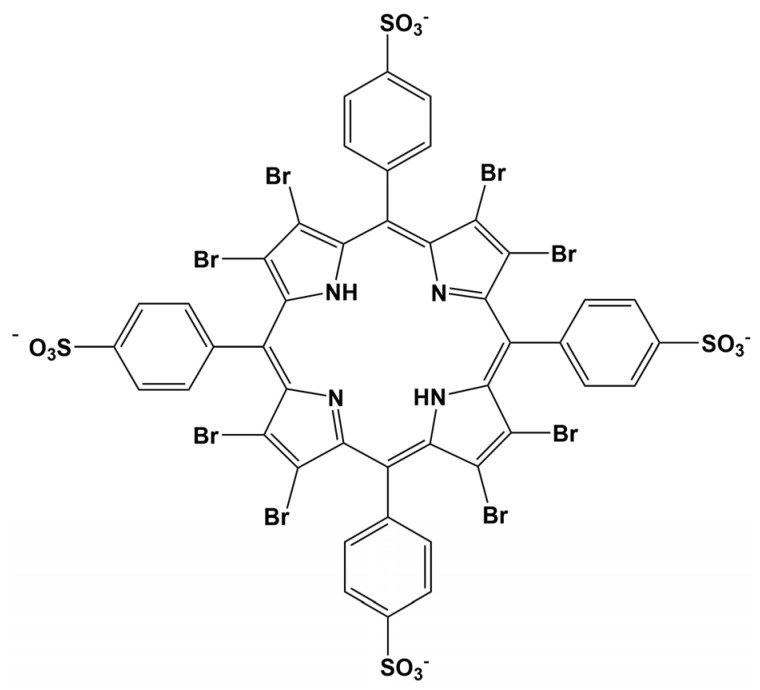
2,3,7,8,12,13,17,18-octabromo-5,10,15,20-tetrakis(4-sulfonatophenyl)porphyrin (Br_8_TPPS_4_).

Among the various factors leading to the formation of J-aggregates, the initial protonation of the macrocycle core with the concomitant distortion of the porphyrin ring is a prerequisite. The addition of two protons to the central nitrogen atoms decreases the overall negative charge, thus decreasing the electrostatic repulsion in the initial step for the onset of dimers. Also, the saddled conformation of the porphine ring is accompanied by the rotation of the *meso* phenyl rings, affording a final structure that is even more planar in comparison with the neutral porphyrin [36]. Considering this point, we thought it of interest to investigate J-aggregate formation starting from a porphyrin that is already strongly distorted in its neutral form. Consequently, we focused our attention on the 2,3,7,8,12,13,17,18-octabromo-5,10,15,20-tetrakis(4-sulfonatophenyl)porphyrin (Br_8_TPPS_4_) (Scheme 1). This compound in its neutral form is highly distorted due to the bulkiness of the bromine substituents in the β-pyrrole positions and the steric clash with the ortho hydrogen atoms of the phenyl groups. This conformation is responsible for a strong enhancement in the rate of metal ion coordination with respect to the planar TPPS_4_ [37]. For this reason, it is quite difficult to investigate the behavior of this compound in neutral solutions, due to its increased propensity, with respect to the unbrominated porphyrin, to take up zinc(II) from environmental sources [38]. To the best of our knowledge, the dimerization process at neutral pH has been investigated [39] and early evidence of J-aggregates under acidic conditions and strong ionic strength has been reported [40]. Here, we describe a kinetic investigation of the formation of these J-aggregates, and depolarized resonant light scattering (RLS) has been applied to get insights into their electronic properties. A theoretical effort using Density Functional Theory (DFT) has been devoted to a simple model of this molecule, aiming to describe the electronic properties of the parent porphyrin, its diacid form and the Raman spectra obtained for the aggregated species deposit.

## 2. Materials and Methods

### 2.1. Materials

The porphyrin 2,3,7,8,12,13,17,18-octabromo-5,10,15,20-tetrakis(4-sulfonatophenyl)porphyrin (Br_8_TPPS_4_) in its acid form was purchased from PorphyChem (France). Sodium chloride and hydrochloric acid were of the highest commercial grade available and were used as received without further purification (Sigma-Aldrich, Milan, Italy). Water used for the experiments was ultra-pure double-distilled from Galenica Senese (Siena, Italy).

Stock solutions of the porphyrin were freshly prepared in water dissolving a small amount of solid porphyrin and stored in the dark. Concentration of the stock solution was determined spectrophotometrically by using ε = 1.41 × 10^5^ cm^−1^ M^−1^ (at 475 nm).

### 2.2. Methods

UV/Vis extinction spectra were measured on a diode array spectrophometer Agilent 8453. To avoid photodamage, all the kinetic experiments were performed by filtering the UV component of the lamp using a Hoya glass type UV-34 filter (Milan, Italy). Circular dichroism (CD) spectra were collected on a Jasco J-710 spectropolarimeter (Milan, Italy). Resonance light scattering (RLS) spectra were acquired on a Jasco FP-750 spectrofluorimeter with a synchronous scan protocol and a right-angle geometry was used to acquire resonance light scattering spectra [41]. Polarizers (Sterling Optics 105 UV) were used to polarize the excitation beam and to analyze the scattered light. The depolarization ratio ρ_v_(90) was measured by applying a correction factor according to the following: ρ_v_(90) = G × I_vH_/I_VV_ (G = I_HV_/I_HH_), where I_VV_ and I_VH_ are the light intensities scattered with vertical and horizontal polarization, respectively.

All the kinetic measurements were acquired by repetitive scanning of the UV/Vis spectra as a function of time. The reagents were mixed in a UV/Vis quartz cuvette that was placed in the thermostated compartment of the spectrophotometer. The temperature was controlled by an external circulating water bath set at 298 K. The kinetic runs were performed adopting a PF protocol: a proper volume of HCl 6M was added to a prediluted porphyrin aqueous solution and the reagents were adequately mixed by inverting the cuvette twice. The extinction data collected at 521 nm as a function of time, *Ext*, were analyzed through a best-fitting procedure applying the following equation [42]:

*Ext* = *Ext_inf_* + (*Ext_0_* − *Ext_inf_*) (1 + (*m* − 1) {*k_0_*t + (*n* + 1)^−1^ (*k_c_*t)*^n^*^+1^})^−1/(*m*−1)^ (1)

where *Ext_0_* (extinction at *t* = 0), *Ext_inf_* (extinction at completion of the aggregation process), *k_c_*, *k_0_*, *m*, and *n* are the parameters to be optimized.

Thin films to be visualized through microscopy were obtained by evaporating aqueous solutions containing J-aggregates on glass cover slides. The morphology of the sample was evaluated using atomic force microscopy measurements acquired using an AFM microscope–NTEGRA-Spectra NT-MDT working in tapping mode. The setup consists of a system that integrates a confocal Raman spectrometer using a solid-state laser operating at 532 nm. The radiation scattered by the sample is collected with a long-working-distance Mitutoyo objective (NA 0.75) and sent to a SOL MS3504I spectrometer equipped with an iDus Andor CCD camera. Raman measurements were performed keeping the laser power at a few microwatts to avoid any damage to the molecules.

### 2.3. Computational Details

The theoretical calculations in this study were performed by means of the Gaussian 09 software package [43] and the output files were visualized with the Gauss View 5.0.8 program [44]. The simplified molecular geometries (Scheme 3), in which the sulfonate groups have been omitted, were fully optimized using the Density Functional Theory (DFT) at the B3LYP (Becke three-parameter Lee-Yang-Parr) combination of functionals [45,46] in conjunction with the 6-31g(d,p) basis set. All calculations were investigated in water solvent by applying the integral equation formalism polarized continuum model (IEF-PCM) [47]. Therefore, the Time-Depending Density Functional Theory (TD-DFT) method [48,49] was used to calculate the UV-vis absorption spectra. The theoretical results were compared with the experimental UV-Vis spectra. The frontier molecular orbitals (FMOs), including HOMOs and LUMOs, and the gap energies were computed based on the optimized geometries to understand the structures and their related electro-optical properties. Vibrational frequencies calculations on the optimized geometries were also performed utilizing the same basis set.

## 3. Results and Discussion

### 3.1. Kinetics of Self-Aggregation

**Figure 1:**
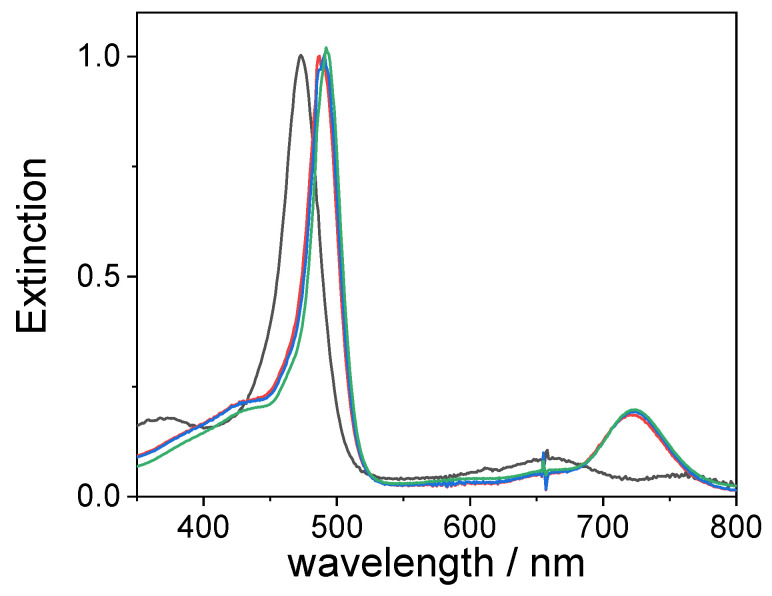
Normalized UV/Vis spectra of Br_8_TPPS_4_^4−^ (pH = 7, black line), H_2_Br_8_TPPS_4_^2−^ ([HCl] = 0.05 M, red line), H_4_Br_8_TPPS_4_ ([HCl] = 2 M, blue line) and H_6_Br_8_TPPS_4_^2+^ (6 M HCl, green line) in aqueous solution ([Br_8_TPPS_4_] = 3 µM).

The free base Br_8_TPPS_4_^4−^ in neutral aqueous solution displays a UV/Vis spectrum dominated by a strong B-band at 475 nm, accompanied by two Q-bands at 660 and 760 nm (Figure 1, black line), in agreement with the literature [39]. Upon addition of HCl up to pH = 1.3, the diacid species H_2_Br_8_TPPS_4_^2−^ forms, characterized by a new strong red-shifted B-band at 489 nm and two Q-bands at 650 (weak) and 724 (medium) nm (Figure 1, red line) [39]. Lowering the pH, the UV/Vis spectrum reveals a very small shift of the B-band (490 nm, Figure 1, blue line) that could be due either to the formation of the neutral zwitterionic species H_4_Br_8_TPPS_4_, in analogy with TPPS_4_ [50], or to a solvatochromic effect. In the presence of 6 M HCl, the spectrum undergoes a further slight shift of the B-band and the Q-bands at lower energies (492 nm, Figure 1, green line), ascribable to the species H_6_Br_8_TPPS_4_^2+^ with four sulphonic acid residues (vide infra). A similar species has been also reported for TPPS_4_ under the same experimental conditions [51].

**Figure 2:**
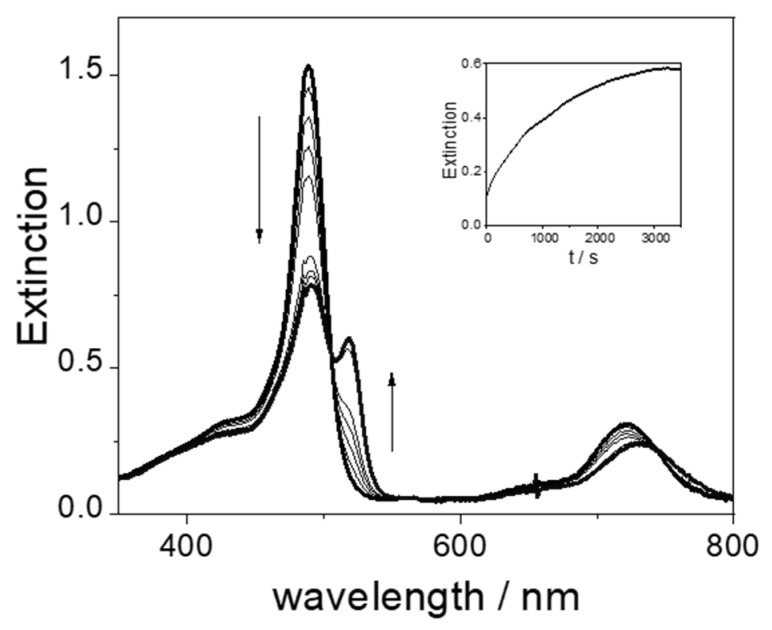
UV/Vis spectral changes for the conversion of the zwitterionic H_4_Br_8_TPPS_4_ into its J-aggregate ([Br_8_TPPS_4_] = 10 μM, [NaCl] = 0.05 M, [HCl] = 0.5 M, T = 298 K). The inset shows the kinetic trace of the absorbance change at 521 nm.

The kinetics for the growth of J-aggregates of TPPS_4_ strongly depend on the total ionic strength and on the mixing protocol [35,50]. We demonstrated that when porphyrin is added as last reagent into a solution containing the acid (*porphyrin last*, PL) the aggregation is fast and the kinetics follow a stretched exponential behavior, leading to J-aggregates that are chiroptically silent. On the contrary, when the acid is added to a prediluted porphyrin solution (*porphyrin first*, PF), the time evolution of the extinction is characterized by sigmoidal profiles and the final aggregates are chiral, as proven by the observation of circular dichroism (CD) bands in their solutions. When a salt is added to further increase the ionic strength, the situation becomes even more complex [35]. Under the experimental conditions reported by Pasternack [40], upon sequential addition of NaCl 0.05 M and HCl 0.5 M to an aqueous solution of Br_8_TPPS_4_, the initially formed diacid or zwitterionic species converts to a new species having a band centered at 521 nm (Figure 2), assigned to the J-aggregate.

**Figure 3:**
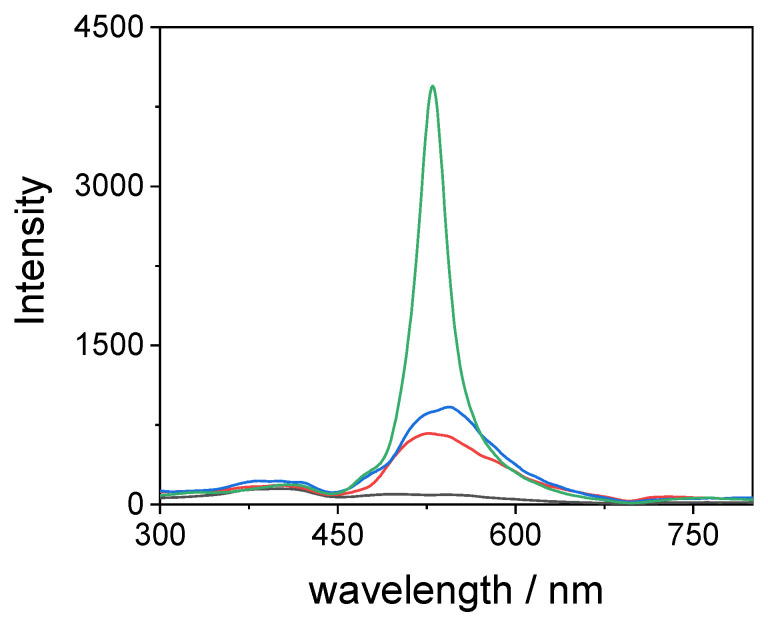
RLS profile for the neat solvent (black line), the neutral Br_8_TPPS_4_ (blue line), its diacid form H_2_Br_8_TPPS_4_^2−^ (red line) and the J-aggregates (green line). Experimental conditions for this latter sample as in Figure 2.

The presence of the aggregated H_4_Br_8_TPPS_4_ is confirmed by the RLS spectra measured on the solution after equilibration that show a resonant peak at 530 nm, red-shifted with respect to the absorption band and strongly enhanced in comparison to the net solvent (Figure 3, green and black lines, respectively). For the sake of comparison, the parent free base porphyrin and its acid forms (Figure 3, blue and red lines, respectively) exhibit much lower intensities and wells corresponding to their absorption bands.

**Figure 4:**
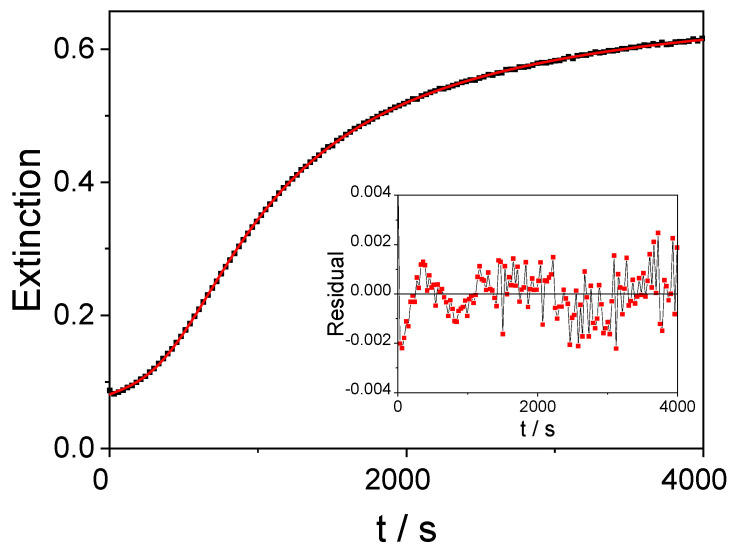
Sigmoidal profile for the time evolution of the extinction measured at 521 nm for the aggregation of H_4_Br_8_TPPS_4_. The red line represents the best-fit curve of the experimental data with Equation (1) (*k*_0_ = (1.44 ± 0.11) × 10^−4^ s^−1^, *k*_c_ = (1.50 ± 0.01) × 10^−3^ s^−1^, *m* = 3.60 ± 0.10, *n* = 2.10 ± 0.07, *R*^2^ = 0.9995). In the inset are the residuals from the best-fitting procedure. Experimental conditions: [Br_8_TPPS_4_] = 10 μM; [HCl] = 1.0 M; PF mixing protocol, T = 298 K.

The kinetic traces collected at the aggregate J-band display an almost exponential behavior with some instability at the end of the process, determining a certain difficulty in reproducing the data. For these reasons, we decided to change the experimental conditions, adopting a PF mixing protocol using HCl only to foster the aggregation process. Figure 4 shows a typical sigmoidal kinetic trace obtained, recording the extinction evolution at 521 nm for this type of experiment. Pasternack developed a kinetic equation (Equation (1)) to treat self-assembling processes in which a slow nucleation stage precedes a fast autocatalytic growth [42]. According to this model, the rate-determining step is the formation of a nucleus containing *m* monomers, followed by an autocatalytic assembling of porphyrins that is controlled by a rate constant *k_c_* and a time exponent *n*, referring to the dimensionality of the process. A non-catalytic pathway is also taken into account by a *k_0_* rate constant. Table 1 collects the relevant kinetic parameters obtained by a best-fitting procedure of the experimental extinction data to Equation (1), when aggregation of Br_8_TPPS_4_ is triggered by different concentration of HCl ([Br_8_TPPS_4_] = 10 μM, PF protocol) or starting with different porphyrin concentration (at [HCl] = 1.0 M).

An inspection of the data reveals that *k_0_* is, on average, an order of magnitude or smaller than *k_c_*. The relative amplitudes of these values impacts on the quite steep profile of the sigmoidal growth, different from those usually observed for the aggregation of TPPS_4_. In the case of this latter porphyrin, a much longer incubation or lag time is present in the kinetic profiles [50]. The number of monomers involved in the rate-determining step of nucleation is 3–4, in agreement with the values reported for the unbrominated porphyrin [50]. The time exponent *n* is in the range 0.8–3.5. For [HCl] < 0.8 M, the kinetics become quite fast, missing the nucleation step, thus preventing a proper analysis of the data. As in the case of other similar systems, we also observed the occurrence of a porphyrin concentration threshold: below this value, either the aggregation process does not occur, or it is slow enough to prevent the possibility of an adequate data collection and analysis. In our case, at [HCl] = 1 M, aggregation is not observed for [Br_8_TPPS_4_] ≤ 6 μM.

**Figure 5:**
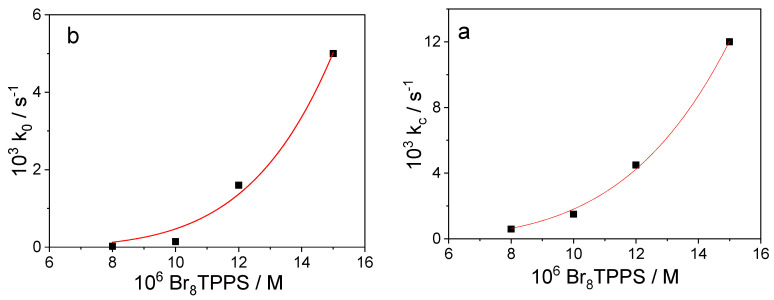
Plot of *k_c_* (**a**) and *k_0_* (**b**) as functions of [Br_8_TPPS_4_] for aggregation of this porphyrin at [HCl] = 1.0 M, T = 298 K. The solid line represents the best-fitting curve to the equations *k_c_* = *a* × [Br_8_TPPS_4_]*^b^* (*a* = (3.81 ± 2.47) × 10^−8^; *b* = 4.67 ± 0.24, *R*^2^ = 0.997) and *k_0_* = *a* × [Br_8_TPPS_4_]*^b^* (*a* = (6.86 ± 15.26) × 10^−10^; *b* = 5.83 ± 0.82, *R*^2^ = 0.989).

The values of *k_c_* as a function of [Br_8_TPPS_4_] (at constant proton concentration, [HCl] = 1.0 M) evidences a monotonically increasing profile (Figure 5a) that can be adequately fitted through the equation *k_c_* = *a* × [Br_8_TPPS_4_]*^b^* (*a* = (3.81 ± 2.47) × 10^−8^; *b* = 4.67 ± 0.24, *R*^2^ = 0.997). This concentration dependence is different from that observed for TPPS_4_, which shows a quadratic behavior [50]. 

**Figure 6:**
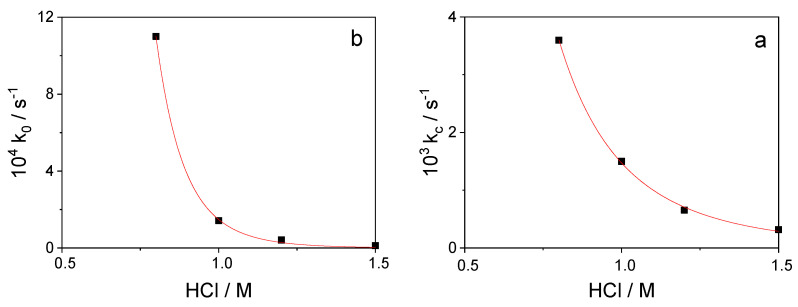
Plot of *k_c_* (**a**) and *k_0_* (**b**) as functions of [HCl] for aggregation of this porphyrin at [Br_8_TPPS_4_] = 10 μM, T = 298 K. The solid line represents the best-fitting curve to the equations *k_c_* = *a* × [HCl]*^b^* (*a* = (1.47 ± 0.03) × 10^−3^; *b* = −4.01 ± 0.10, *R*^2^ = 0.9993) and *k_0_* = *a* × [HCl]*^b^* (*a* = (1.48 ± 0.11) × 10^−4^; *b* = −8.97 ± 0.34, *R* = 0.999).

Upon increasing the proton concentration, the *k_c_* values decrease (Figure 6a) according to the equation *k_c_* = *a* × [HCl]*^b^* (*a* = (1.47 ± 0.03) × 10^−3^; *b* = −4.01 ± 0.10, *R*^2^ = 0.9993). This behavior is reminiscent of what is observed for TPPS_4_: the pH range useful for the growth of the J-aggregates is limited in the lower region by a minimum threshold acid concentration for activating the self-aggregation process, and in the upper region by the formation of H_6_Br_8_TPPS_4_^2+^. This species, being all the sulphonate groups fully protonated, lacks the specific electrostatic favorable contacts to interact with the protonated nitrogen atoms of the porphyrin core. The patterns exhibited by the *k_0_* values are reported in Figure 5b and Figure 6b and they are similar to those of the catalyzed rate constants (*k_0_* = *a* × [ Br_8_TPPS_4_]*^b^* (*a* = (6.86 ± 15.26) × 10^−10^; *b* = 5.83 ± 0.82, *R* = 0.989; *k_0_* = *a* × [HCl]*^b^* (*a* = (1.48 ± 0.11) × 10^−4^; *b* = −8.97 ± 0.34, *R* = 0.999)).

**Figure 7:**
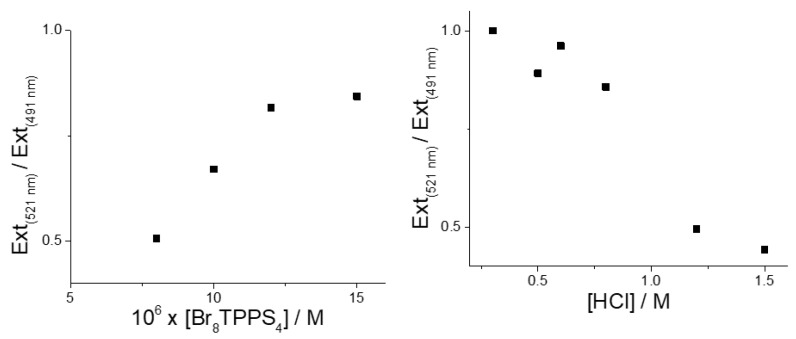
Plot of the ratio of the extinction values at 521 and 491 nm after completion of the aggregation process as a function of porphyrin (**left**) and hydrochloric acid concentration (**right**).

The relative quantity of J-aggregates obtained with respect to the residual monomer in solution has been estimated from the ratio between the extinction values measured at 521 and 491 nm, respectively. Plots of this ratio as a function of [Br_8_TPPS_4_] and [HCl] are reported in Figure 7, indicating that the formation of the aggregate is favored on increasing the porphyrin concentration and decreasing the acid concentration. 

Differently from J-aggregates of TPPS_4_ obtained with the same protocol, the aggregates of the investigated porphyrin do not evidence any induced CD band, suggesting absence of chirality or formation of racemic mixtures in the absence of chiral inducers. Even in the presence of L-tartaric acid 0.1 M, fostering aggregation with 1 M HCl, no chirality transfer has been observed.

### 3.2. Depolarized Resonance Light Scattering Investigations

**Figure 8:**
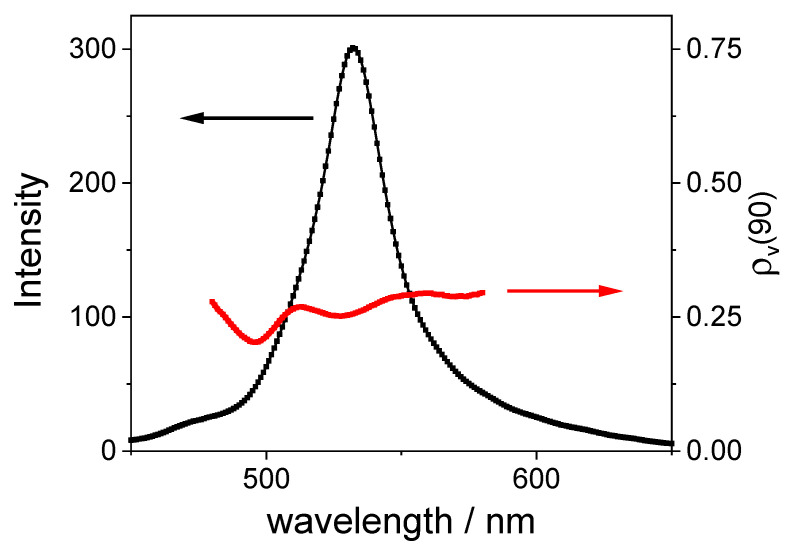
RLS spectrum (black line) and depolarization ratio ρ_v_(90) (red line) of J-aggregates of H_4_Br_8_TPPS_4_ ([Br_8_TPPS_4_] = 10 µM; [HCl] = 1 M; T = 298 K).

Resonance light scattering is a powerful technique to investigate aggregated chromophores in solution. A strong enhancement of the scattered light intensity occurs when a quite large number of monomeric units are assembled (N > 25) [41], and strong electronic coupling is operative. Parkash et al. theoretically described the dependence of the depolarization ratio from RLS measurements in order to obtain geometrical information on the chromophore arrangement in the aggregate [52]. The depolarization ratio ρ_v_(90) can be calculated using linearly polarized light as the exciting source, detecting the scattered light at 90° with respect to the incident beam and adopting an orthogonal linear polarizer. It is defined as I_VH_/I_VV_, where I_VH_ is the intensity of the scattered light detected with horizontal polarization and I_VV_ that detected with vertical polarization. The quantum mechanical model developed for different types of geometrical arrangements can be applied to chromophores slipped by an angle φ with respect to the axis connecting their centers or even to cylindrical aggregates. Figure 8 displays the RLS spectrum measured on J-aggregates of H_4_Br_8_TPPS_4_ (black line) together with the depolarization ratio measured in the band profile (red line). An average value ρ_v_(90) ≈ 0.265 can be obtained, and from the theoretical dependence of ρ_v_(90) on the slip angle [52], we can estimate a value of φ ≈ 20° in the J-aggregate. 

**Scheme 2:**
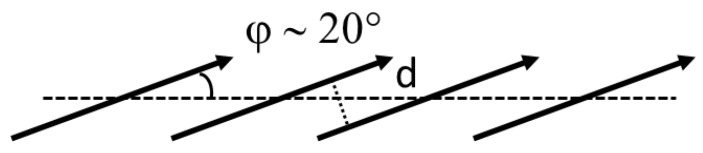
Geometrical sketch of the electronic transition moment arrangement inside the J-aggregate from depolarized light-scattering measurements.

Scheme 2 reports a model for the spatial disposition of the electronic transition moments of the chromophores, where *d* is the distance between adjacent porphyrin planes.

Further information on the electronic properties of these J-aggregates can be obtained from an analysis of the J-band linewidth. In J-aggregates, this band is usually quite sharp due to exchange narrowing. Knapp applied a perturbation treatment to describe the lineshapes of exciton spectra in linear aggregates [16]. According to this model, the linewidth corresponding to an aggregate containing *N* strongly coupled monomers, *W*(N), is related to the linewidth of the non-interacting monomer, *W*(0), by the equation *W*(*N*) = (*W*(0) × *N*^−1/2^)(2 *ln* 2)^1/2^. Deconvolution of the UV/Vis spectra for the monomer and the J-aggregate band afforded *W*(0) = 875 cm^−1^ and *W*(*N*) = 463 cm^−1^, respectively. From these values, we are able to estimate *N*~5 as the spectroscopic aggregation number. In J-aggregates of TPPS_4_, Koti et al. have calculated *N* ranging between 5 and 13 [53]. Assuming a linear arrangement of the chromophores, the coherence length, *L*, can be estimated as (*N* + 1) times the radius of a single monomer. From molecular modeling, this latter is estimated at about 1 nm, thus leading to a value of *L*~6 nm.

**Figure 9:**
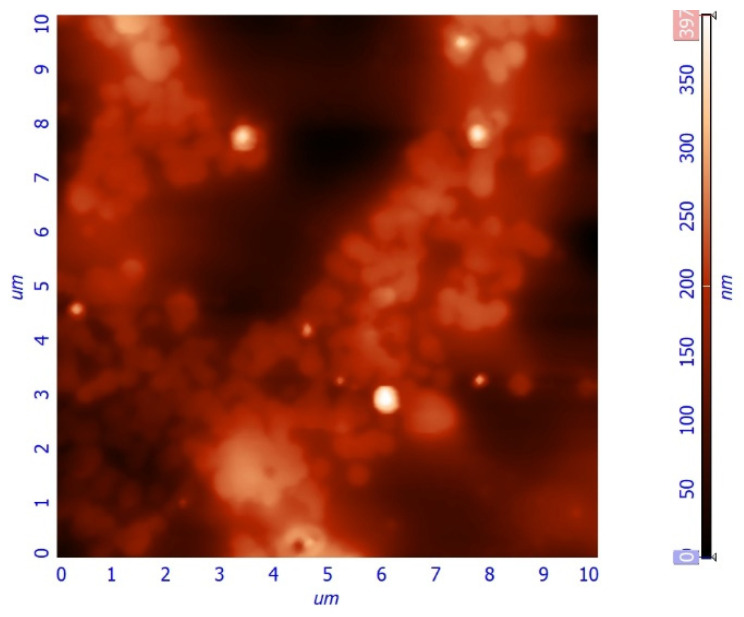
AFM image of J-aggregates of H_4_Br_8_TPPS_4_ deposited on glass cover slides by solvent evaporation at room temperature.

J-aggregates of the title porphyrin have been deposited on glass cover slides by slow evaporation of the solvent from equilibrated solutions. Atomic force microscopy (AFM) has been used to study the morphology of these aggregates. Figure 9 reveals the presence of a population of spherical or oblate aggregates with an average length of 130 nm. This experimental evidence agrees with dynamic light scattering measurements on the same samples in solutions where an average diameter of 150 nm has been calculated (PDI = 0.51). It is interesting to point out that the J-aggregates of Br_8_TPPS_4_ exhibit a bathochromic shift of about 30 nm, much smaller with respect to that occurring in J-aggregates of TPPS_4_ (55 nm). This experimental finding is in line with the different geometrical arrangement and the extent of electronic coupling.

### 3.3. Theoretical Calculations

#### 3.3.1. Molecular Structure and Electronic Properties

**Scheme 3:**
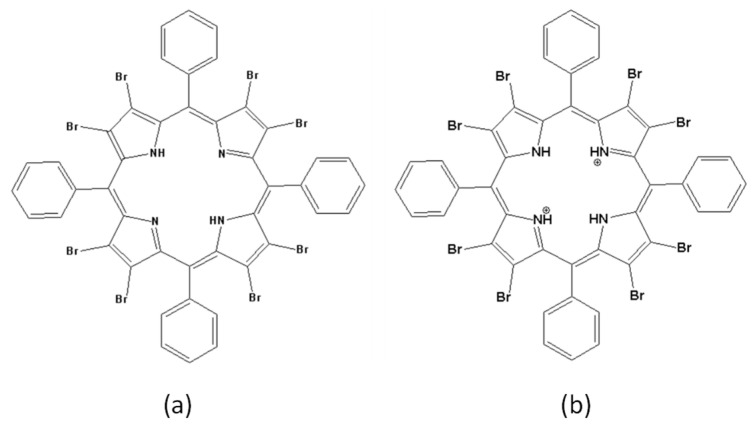
Molecular structure of two simplified compounds used in the theoretical analysis as models for the free base porphyrin Br_8_TPP (**a**) and its diacid species Br_8_TPPH_2_^2+^ (**b**).

DFT method was exploited to investigate our system. In order to simplify the calculations, we used β-brominated-*meso*-tetraphenylporphyrin and its diacid form (Br_8_TPP and Br_8_TPPH_2_^2+^ in Scheme 3) to mimic Br_8_TPPS_4_^4−^ and H_2_Br_8_TPPS_4_^2−^, respectively. 

**Figure 10:**
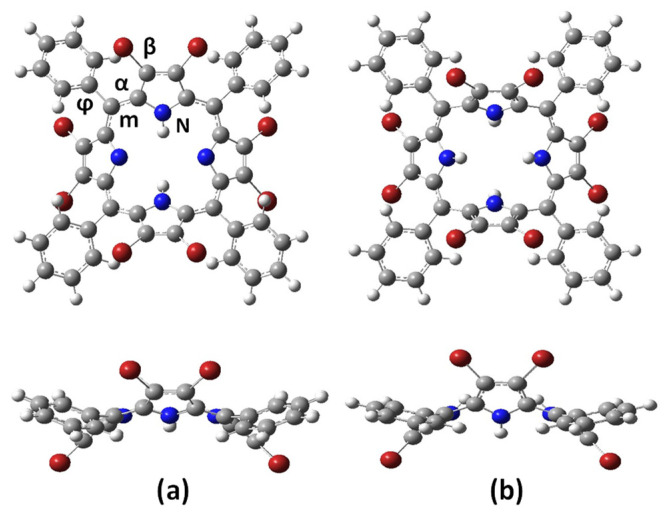
Optimized structures of Br_8_TPP (**a**) and H_2_Br_8_TPP^2+^ (**b**) in top and side view.

The studied structures were optimized at the B3LYP/6-31g(d,p) level of theory to obtain the lowest energy configuration. The optimized ground state geometries (top and side views) of Br_8_TPP and H_2_Br_8_TPP^2+^ are shown in Figure 10. 

The computed molecular parameters including bond lengths, bond angles and dihedral angles are summarized in Table 2.

Both the optimized geometries display a non-planar saddle shape conformation because of steric hindrance between the β-bromo and meso-phenyl groups, which leads to two pyrroles pointing up and two pyrroles pointing down with respect to the porphyrin mean plane [54]. The protonation of the inner nitrogen atoms induces relevant structural changes. According to Table 2, the meso-phenyl groups are oriented at a tilt angle of approximately 51° for the unprotonated structure and approximately 41° for the diprotonated structure. The distances between bonds are different. Specifically, the C_α_–C_β_ distances have decreased by 0.04 Å. On the other hand, the C_α_–C_m_ and C_β_–C_β_ bond has increased by 0.03–0.05 Å. In addition, the protonation of the porphyrin core causes an increase of the hydrogen bond NH…HN (≈0.8 Å), which results in a significant deviation from the unprotonated structure of about 25°.

**Figure 11:**
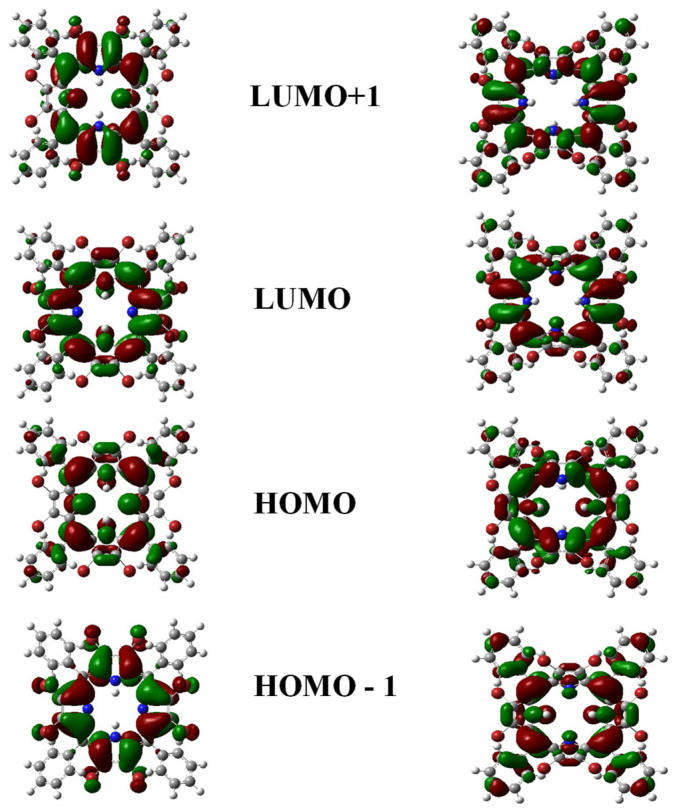
Graphical presentation of selected molecular orbitals of Br_8_TPP (**left**) and H_2_Br_8_TPP^2+^ (**right**).

To get insights into the electronic properties of these molecular systems and the changes induced by the protonation, we investigated the composition and energy levels of the molecular orbitals (MOs). The Highest Occupied Molecular Orbital (HOMO) describes the electron-donating capacity of the molecule, while the Lowest Unoccupied Molecular Orbital (LUMO) energy characterizes its electron-withdrawing capability [55]. The gap energy, E_g_, represents the difference between E_HOMO_ and E_LUMO_. The E_g_ value is essential for assessing the molecular chemical stability and electron conductivity, making it a crucial factor in understanding molecular electrical transport properties [55]. The graphical presentation of HOMO-1, HOMO, LUMO and LUMO + 1 for Br_8_TPP and H_2_Br_8_TPP^2+^ are depicted in Figure 11.

It is worth mentioning that the diprotonated structure exhibits lower energy levels for both the HOMO and LUMO compared to the unprotonated structure. This result leads to a smaller gap for the diprotonated structure (E_g_ (Br_8_TPP) = 2.28 eV, E_g_ (H_2_Br_8_TPP^2+^) = 1.71 eV), indicating that H_2_Br_8_TPP^2+^ is more stable than Br_8_TPP.

#### 3.3.2. UV-Vis Absorption Spectra Analysis

To investigate the impact of protonation on the optical absorption properties, we exploited the TD-DFT method at the B3LYP/6-31g(d,p) level of theory. 

**Figure 12:**
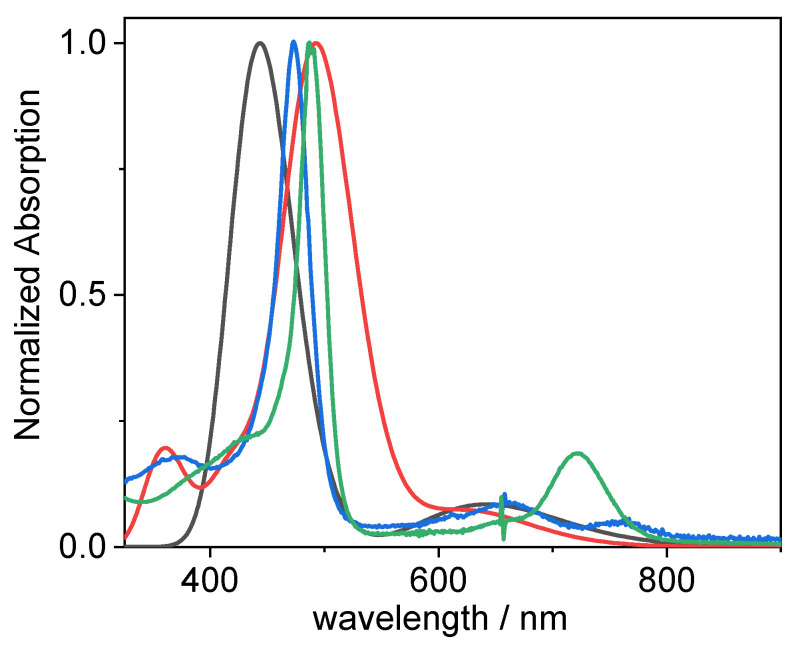
Comparison of experimental UV-Vis absorption spectra of Br_8_TPPS_4_^4−^ (blue line) and H_2_Br_8_TPPS_4_^2−^ (green line), l and calculated ones (6-31g(d,p) basis set) of Br_8_TPP (black line) and H_2_Br_8_TPP^2+^ (red line), in water.

The UV-vis spectra are displayed in Figure 12 and the corresponding calculated data are summarized in Table 3.

The absorption spectra evidence two prominent charge transfer bands. The first ones, referred to as B-bands, are observed at 446 nm and 492 nm for Br_8_TPP and H_2_Br_8_TPP^2+^, respectively, with oscillator strengths of 1.35 and 1.55. These absorption bands are attributed to S_0_ → S_2_ transitions, originating mainly from H-1→LUMO (73%) transition. The second bands (Q-bands) are found at approximately 627 nm and 672 nm, and are attributed to S_0_ → S_1_ transitions, primarily originating from the HOMO → LUMO (80%) and the HOMO → L + 1 (74%). The B-band exhibited a bathochromic shift, moving from 446 nm in the starting Br_8_TPP porphyrin to 492 nm in its diacid species. This red shift by 46 nm is in agreement with a substantial change of the electronic properties and/or structure of the molecule due to protonation of the central core.

Figure 12 reports a comparison between the experimental and the calculated UV/Vis absorption spectra. Considering that the theoretical spectra refer to different model porphyrins (sulphonate groups are missing), we observe that the general pattern of behavior is similar, even if the red shift of the experimental spectra is less than that of the theoretical one (14 vs. 46 nm).

#### 3.3.3. Raman Spectra

**Figure 13:**
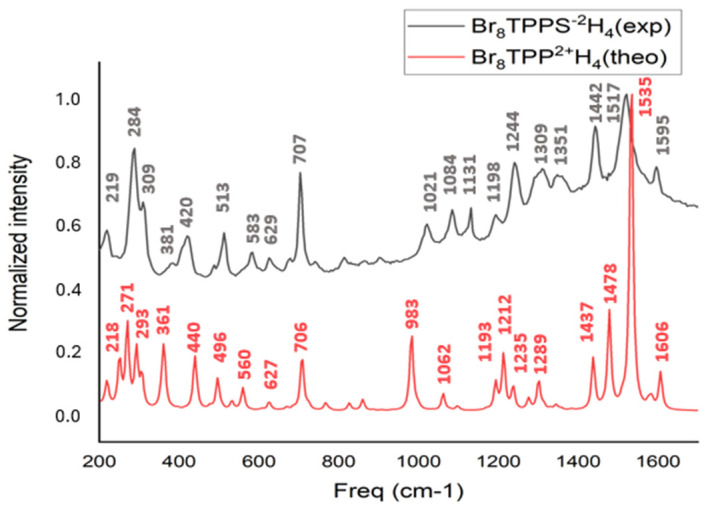
Experimental Raman spectrum of J-aggregated Br_8_TPPS_4_ (**top**) and theoretical spectrum of H_2_Br_8_TPP^2+^ (**bottom**).

The Raman spectra of Br_8_TPPS_4_ J-aggregates were recorded by studying a thin film obtained by slowly evaporating the solvent on glass coverslips (Figure 13, red line). The theoretical Raman spectrum of H_2_Br_8_TPP^2+^ has been calculated and it is reported for comparison (Figure 13, black line). As it is possible to observe from an inspection of these spectra, numerous Raman peaks are present in the region from 200 to 1600 cm^−1^, having strong to weak intensities. When analyzing the measured and computed data, it is evident that the Raman patterns in the spectra of both molecules closely resemble each other. However, there are significant frequency shifts in certain Raman peak positions, and some peaks are absent.

Based on the experimental results shown in Table 4 and the results of the quantum-chemical calculations, the low-frequency part of the Raman spectrum shows two significant peaks: one at 271 cm^−1^, which corresponds to out-of-plane twisting [56], and another at 293 cm^−1^, which is connected to the in-plane breathing motion of the porphyrin ring [57]. These predictions are in agreement with the experimental values of 284 cm^−1^ and 309 cm^−1^. The intermediate peak observed at 361 cm^−1^ corresponds to the breathing motion of whole molecule, involving the stretching of ν(C_β_–Br) bonds. The weak peaks at 496, 560 and 627 cm^−1^ are related to the out-of-plane translational motion of the pyrrole rings, leading to the expansion of the porphyrin core along the NH–NH direction. Another intense band in the low-frequency range occurs at 706 cm^−1^, attributable to stretching of (Cα–NH) bonds and in-plane bending of (NH–Cα–Cm), with a measured frequency of 707 cm^−1^. The Raman peaks observed between 950–1065 cm^−1^ can be attributed to the protonation of the porphyrin core, leading to saddle-like distortions in its structure. The proton addition increases the flexibility of the rocking motion of N–H bonds by reducing the repulsive interaction or steric effects between these hydrogen atoms [58].

The influence of the sulfonate group becomes evident within the range of 1060 to 1200 cm^−1^, and their peaks are obviously missing in the theoretical spectra within this region. Moreover, the strongest peak appearing at 1212 cm^−1^ is mainly due to the stretching of C_ϕ_–C_m_ bonds, along with the symmetric stretching of C_α_–N(H)–C_α_ and weak stretching of C_β_–C_β_ bonds. The remaining medium peaks in the range 1250–1400 cm^−1^ primarily arise from out-of-plane bending of the phenyl ring, including symmetric stretching of C_α_–C_m_–C_ϕ_ bonds. The most prominent Raman bands observed at 1517 (experimental) and 1535 cm^−1^ (theoretical) arises from vibrational movements, specifically the stretching of C_β_–C_β_ bonds and the symmetric stretching of C_α_–C_m_–C_α_ bonds. These vibrational modes ultimately induce a bending deformation in the C_α_–N(H)–C_α_ bonds. Additionally, the Raman peak detected at 1593 cm^−1^ corresponds closely to the calculated value of 1606 cm^−1^, and it is attributed to the stretching of C–C bonds (ν(C–C)) within the phenyl rings.

In general, the comparison demonstrates a strong correspondence between the calculated frequencies and the experimental data, thereby confirming the validity of the theoretical vibrational analysis.

## 4. Conclusions

The highly non-planar Br_8_TPPS_4_ porphyrin is able to form J-aggregates in aqueous solutions, under quite strong acidic conditions. The kinetics show dependence on the mixing protocols, and when a PF protocol is used, an early nucleation stage followed by a quite rapid growth is observed. In any case, the derived rate constants for the catalytic self-assembling process behave differently with respect to TPPS_4_, exhibiting a much stronger porphyrin concentration dependence. These species do not exhibit spontaneous symmetry breaking, nor is chirality transferred through chiral chemical templates. The internal arrangement of the chromophores is in line with an edge-to-edge geometrical disposition, with the porphyrins strongly electronically coupled and consistently tilted with respect to the line connecting the centroids of the molecules. The exciton is delocalized over an average of five monomeric units and the coherence length is about 6 nm, that is, roughly 10% of the entire length of a single aggregate. AFM reveals that these J-aggregates have an almost globular morphology, quite different from the nanotubular structure exhibited by the unbrominated porphyrin TPPS_4_. DFT and TD-DFT calculations on simplified models gave further insights into the structural and electronic changes caused by protonation of the central nitrogen atoms and were successful in describing the vibrational Raman spectra of these nanoaggregates.

The J-aggregate here described represents a new example, adding to the series of supramolecular self-assembled structures formed by porphyrins. Preliminary experiments have pointed out the extreme reactivity of the free base porphyrin to Zn^2+^, suggesting that, as in other J-aggregated systems [19,59], the size of these nano-assemblies could be potentially modulated by the presence of specific metal ions. We expect that, due to the important differences in electronic and structural behavior with respect to the TPPS_4_ porphyrin, and especially in terms of reactivity to metal ions, e.g., Li^+^, Cd^2+^, Hg^2+^ and Zn^2+^ [37,38], these systems could be potentially useful for a variety of applications, including the development of sensors.

## Figures and Tables

**Table 1:** Values for the kinetic parameters *k*_0_, *k_c_*, *m* and *n* calculated through a best-fitting procedure of extinction data to Equation (1), for the acid-induced J-aggregation of H_4_Br_8_TPPS_4_ ([Br_8_TPPS_4_] = 8–15 μM; [HCl] = 0.8–1.5 M; PF mixing protocol, T = 298 K).

10^6^ [Br_8_TPPS_4_]/M	[HCl]/M	10^4^ *k*_0_/s^−1^	10^3^ *k*_c_/s^−1^	*m*	*n*
8	1	0.260 ± 0.020	0.594 ± 0.001	3.67 ± 0.06	3.46 ± 0.06
10	1	1.44 ± 0.11	1.50 ± 0.01	3.60 ± 0.10	2.10 ± 0.07
12	1	10.6 ± 0.7	4.55 ± 0.07	4.53 ± 0.16	1.84 ± 0.10
15	1	49.0 ± 0.5	12.0 ± 0.8	6.50 ± 0.67	1.84 ± 0.22
10	0.8	11.0 ± 2.3	3.70 ± 0.30	2.94 ± 0.28	0.862 ± 0.191
10	1.2	0.420 ± 0.020	0.657 ± 0.002	4.00 ± 0.07	2.91 ± 0.05
10	1.5	0.130 ± 0.011	0.320 ± 0.001	3.30 ± 0.05	2.98 ± 0.04

**Table 2:** Selected optimized structural parameters for Br_8_TPP and H_2_Br_8_TPP^2+^ from DFT calculations.

	Br_8_TPP	H_2_Br_8_TPP^2+^
Bond distances (Å)		
C_β_–C_β_	1.39	1.42
C_α_–C_β_	1.44	1.40
C_α_–N	1.39	1.39
C_α_–C_m_	1.42	1.47
C_m_–C_ϕ_	1.48	1.48
C_β_–Br	1.93	1.93
N–H_N_	1.01	1.01
Bond angles (°)		
C_α_–C_m_–C_α_	121.2	118.9
C_α_–N–C_α_	112.3	111.7
C_α_–C_β_–C_β_	108.5	108.3
N–C_α_–C_β_	105.0	105.7
N–C_α_–C_m_	123.5	122.0
C_β_–C_β_–Br	124.1	125.7
C_α_–C_m_–C_ϕ_	119.1	117.6
C_α_–N–H_N_	123.7	123.8
Dihedral angles (°)		
N–C_α_–C_β_–Br	−167.6	−176.0
C_m_–C_α_–C_β_–C_β_	−172.1	−174.6
C_α_–C_β_–C_β_–C_α_	0	0
N–C_α_–C_β_–C_β_	3.5	0.8
H_N_–N–C_α_–C_β_	174.4	−173.6
N–C_α_–C_m_–C_ϕ_	158.8	−154.3
N–C_α_–C_m_–C_α_	25.5	54.8
C_α_–C_m_–C_α_–C_β_	−159.5	−130.3
C_ϕ_–C_m_–C_α_–C_β_	20.6	47.0

**Table 3:** Absorption spectra data for Br_8_TPP and H_2_Br_8_TPP^2+^ calculated at TD-DFT//B3LYP/6-31g(d,p) level in water.

Structure	λ_max_ (nm)	Osc. Strength	Major Contribs (%)	Minor Contribs (%)
Br_8_TPP	446	1.35	H-1 → LUMO (73%), HOMO → L + 1 (23%)	H-8 → L + 1 (2%)
	672	0.11	HOMO → LUMO (80%), H-1 → L + 1 (19%)	_____
H_2_Br_8_TPP^2+^	492	1.55	H-1 → LUMO (73%), HOMO → L + 1 (26%)	H-1 → LUMO (73%), HOMO → L + 1 (26%)
	627	0.10	HOMO → L + 1 (74%), H-1 → LUMO (26%)	_____

**Table 4:** Vibrational assignments of the experimental Raman band of J-aggregated Br_8_TPPS_4_, in comparison with the calculated results for H_2_Br_8_TPP^2+^.

ν_exp_ (cm^−1^)	ν_theo_ (cm^−1^)	Vibrational Assignments
219	218	δ (phenyl)
284	271	γ (porph ring), δ (phenyl)
309	293	Breath (porph ring), δ (phenyl)
381	361	Breath (whole molecule), ν(C_β_–Br)
420	440	ν_s_ (C_β_–C_α_–C_m_), ν(C_β_–Br)
513	496	γ (NH), γ (phenyl)
583	560	γ (NH),
629	627	γ (NH), δ (phenyl)
707	706	δ(NH–C_α_–C_m_), ν(C_α_–NH)
1021	983	ν (C_β_–C_α_), ν_s_(C_α_–NH–C_α_), δ (phenyl)
1084	1062	ν (C_β_–C_α_), ν(C_m_–C_α_), γ (phenyl)
1131	—	ν_as_ (O–S–O)
1198	1193	γ (C_phenyl_–H)
1244	1212	ν (C_ϕ_–C_m_), ν_s_ (C_α_–NH–C_α_), ν (C_β_–C_β_)
1309	1289	γ (Phenyl)
1351	—	ν_s_ (C_α_–C_m_–C_ϕ_), γ (Phenyl)
1442	1437	ν (C_β_–C_β_), ν_s_ (C_β_–C_α_–C_m_)
1517	1535	ν (C_β_–C_β_), ν_s_ (C_α_–C_m_–C_α_), θ(C_α_–NH–C_α_)
1595	1606	Ν (C_phenyl_–C_phenyl_)

*ν*, *δ* and *γ* denote stretching, in-plane bending and out-of-plane bending modes, respectively.

## Data Availability

Data are available upon request.
